# Conference Calendar

**DOI:** 10.1002/cfg.330

**Published:** 2003-10


					Comparative and Functional Genomics
Comp Funct Genom 2003; 4: 558-559.
Published online in Wiley InterScience (www.interscience.wiley.com). DOI: 10.1002/cfg.330
Conference Calendar
January-March 2004
Animal models
KEY -- Natural Variation and Quantitative
Genetics in Model Organisms, Jan 8-13
http://www.symposia.com/Meetings/View
Meetings.cfm?MeetingID=671
45th Annual Drosophila Research Conference,
Mar 24-28
http://flybase.bio.indiana.edu/docs/news/
announcements/meetings/
Bacteria
KEY -- Bacterial Chromosomes, Feb 7-12
http://www.keystonesymposia.org/Meetings/
ViewMeetings.cfm?MeetingID=699
Bioinformatics
Pacific Symposium on Biocomputing (PSB
2004), Jan 6-10
http://psb.stanford.edu/
O'Reilly Life Science Informatics Conference,
Feb 9-12
http://conferences.oreillynet.com/Isi2004/
GRC -- Genomics & Structural/Evolutionary
Bioinformatics, Feb 15-20
http://www.grc.uri.edu/04sched.htm#GRC
8th Annual International Conference on
Research in Computational Molecular Biology
(RECOMB 2004), Mar 27-31
http://recomb04.sdsc.edu/
Evolution/comparative genomics
Society of Integrative and Comparative Biology
Annual Meeting, Jan 4-8
http://www.sicb.org/meetings/2004/
GRC -- Molecular Evolution, Feb 1-6
http://www.grc.uri.edu/04sched.htm#GRC
General
KEY -- Emerging Mechanisms of Epigenetic
Regulation, Jan 21-26
http://www.symposia.com/Meetings/ViewMeet
ings.cfm?MeetingID=700
Multigenomes
Plant and Animal Genome XII Conference,
Jan 10-14
http://www.intl-pag.org/
Pharmacogenomics
KEY -- Human Genome Sequence Variation
and the Inherited Basis of Common Disease,
Jan 8-13
http://www.symposia.com/Meetings/View
Meetings.cfm?MeetingID=670
AACR -- Oncogenomics 2004: Dissecting
Cancer through Genome Research, Jan 21-25
http://www.aacr.org/4200m.asp#Jan04
KEY -- How Will Genomic Biomarkers Impact
Drug Discovery and Clinical Practice?
Jan 26-30
http://www.symposia.com/Meetings/View
Meetings.cfm?MeetingID=705
CHI -- High Content Analysis, Jan 29-30
http://www.healthtech.com/2004/hca/
IBC -- 4th International Conference
InfoTechPharma 2004, Feb 2-5
http://www.ibc-lifesci.com/forthcoming/forth
new/html/introduction.asp?page=it
Copyright  2003 John Wiley & Sons, Ltd.
Conference Calendar 559
IBC -- Drug Discovery Technology, Mar 8-11
http://www.drugdisc.com/europe/
IBC -- Screentech World Summit, Mar 21-25
http://www.lifesciencesinfo.com/screentech/
CHI -- Molecular Medicine Tri-Conference,
Mar 23-26
http://www.chiMolecularMed.com
Plants
KEY -- Comparative Genomics of Plants, Mar
4-9
http://www.keystonesymposia.org/Meetings/
ViewMeetings.cfm?MeetingID=688
Proteomics
CHI -- PEPTALK: The Protein Information
Week, Jan 12-15
http://www.chi-peptalk.com/
ABRF -- Integrating Technologies in
Proteomics and Genomics, Feb 28-March 2
http://mercury.faseb.org/abrf2004/default.htm
Structural genomics
Biophysics Society 48th Annual Meeting, Feb
14-18
http://www.biophysics.org/annmtg/
Systems biology
KEY -- Mass Spectrometry in Systems Biology,
Feb 14-19
http://www.keystonesymposia.org/Meetings/
ViewMeetings.cfm?MeetingID=704
Transcriptomics
International qPCR Symposium and Application
Workshop: Transcriptomics, Clinical
Diagnostics and Gene Quantification, Mar 3-6
http://www.wzw.tum.de/gene-quantification/
qpcr2004/index.shtml
Yeasts and fungi
ASM -- Candida and Candidiasis, Mar 18-22
http://www.asm.org/Meetings/index.asp?bid
=17814
Physiology of Yeasts and Filamentous Fungi
(PYFF2), March 24-28
http://pyff2.insa-tlse.fr/
SGM -- 154th Meeting, Mar 29-Apr 2
http://www./sgm.ac.uk/meetings/MTGPAGES/
Bath.cfm
Biotech industry
2nd International Bio Trends, Feb 16-18
http://www.biotrends.de/biotrends/
Key
AACR -- American Association of Cancer Re-
searchers: http://www.aacr.org
ABR -- Association of Biomolecular Resource
Facilities: http://www.abrf.org
ASMI -- American Society for Microbiology:
http://www.asm.org
CHI -- Cambridge Healthtech Institute: http://
www.healthtech.com
GRC -- Gordon Research Conferences: http://
www.grc.uri.edu/
IBC -- IBC Conferences: http://www.ibc-life
sci.com
KEY -- Keystone Symposia: http://www.sympo
sia.com
SGM -- Society for General Microbiology: http://
www.sgm.ac.uk
Copyright  2003 John Wiley & Sons, Ltd. Comp Funct Genom 2003; 4: 558-559.

				

